# Buschke-Löwenstein Tumour: Surgical Management and Literature Review of an Unusual Disease

**DOI:** 10.7759/cureus.85639

**Published:** 2025-06-09

**Authors:** Mei-Ting Chen, Ferdinand Ong, Kim-Chi Phan-Thien

**Affiliations:** 1 Department of Surgery, Redcliffe Hospital, Redcliffe, AUS; 2 Department of Surgery, St. George Hospital, Kogarah, AUS; 3 Department of Surgery, St George Hospital, Kogarah, AUS

**Keywords:** ano rectal diseases, buschke-löwenstein tumor, colorectal disease, diverting colostomy, perineal reconstruction

## Abstract

Buschke-Löwenstein tumours (BLTs) are rare, sexually transmitted lesions associated with human papillomavirus (HPV) infections that primarily affect the perineal and genito-anal regions. Surgical excision is the standard treatment; however, management is often complicated by the tumour's local invasiveness, disease progression, variable treatment response, and high recurrence rates. Wound healing is especially challenging due to the proximity to perineal structures, increasing the risk of contamination and infection. We present the case of a man in his 50s with a large, circumferential anal BLT and a background of immunosuppression from prednisolone and mycophenolate treatment. This case highlights the complexities of managing BLTs and demonstrates surgical strategies, including reconstruction and diverting colostomy, to optimise both cosmetic and functional outcomes.

## Introduction

Buschke-Löwenstein tumours (BLTs), also known as giant condyloma acuminata, are rare sexually transmitted lesions commonly linked to low-risk human papillomavirus (HPV) genotypes 6 or 11 and, less frequently, high-risk genotypes 16 or 18 [1]. These tumours have an estimated prevalence of 0.1% in the general population and predominantly affect men [2]. Other risk factors include immunodeficiencies, poor hygiene, multiple sexual partners, chronic genital infections, and is strongly associated with HIV infection [1,2]. BLTs typically occur on the vulva, penis, groin, perineum, anus, perianal skin, or suprapubic area [3]. They are often described as exophytic, ulcerative, cauliflower-like masses that can infiltrate adjacent tissues [2,4]. Surgical excision is the treatment of choice [4]. While the diagnostic process is usually uncomplicated due to its characteristic appearance, wound care and high recurrence rates often pose challenges to its management [5]. Although its histological features appear benign, BLT is locally destructive and has the potential for malignant transformation [1,2]. 

## Case presentation

A 57-year-old male patient presented with an incidental finding of a large perianal condyloma acuminata during the treatment for newly diagnosed focal segmental glomerulosclerosis (FSGS). He initially presented with lethargy and was found to have an acute renal injury, with raised creatinine and a decline in eGFR (Table 1). The remaining blood tests, including white blood cells and electrolytes, were unremarkable. His medical history included insulin-dependent type II diabetes mellitus, peripheral vascular disease, non-Hodgkin’s lymphoma (in remission), and hypertension. The sexually transmitted infection (STI) screen including HIV was negative on admission.

**Table 1:** Summary of blood test results that demonstrate pre-operative optimisation. eGFR: estimated glomerular filtration rate; HbA1C: glycated haemoglobin.

Parameters (reference range/ unit)	On admission	Pre-operation
White blood cell (3.5-11.0) x 10^9^/L	7.5	8.6
eGFR (>90 mL/min/1.732 m^2^)	18	65
Creatinine (36-73 µmol/L)	317	109
Random glucose (3.0-7.8 mmol/L)	7.8	6.8
HbA1C (3.5-6.0%)		6.4

**Figure 1:**
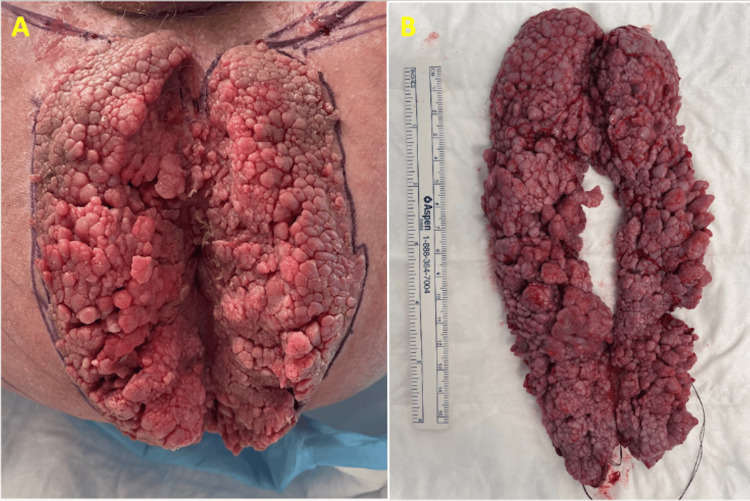
Photographs of the (A) Buschke-Löwenstein tumour with sparing of the anal canal centrally and (B) the excised specimen.

Clinical examination revealed a 25-cm circumferential mass of anal condyloma acuminata with sparing of the immediate perianal skin (Figure 1). A colonoscopy identified small tubular adenomas and diverticulosis.

The Buschke-Löwenstein tumour required operative excision and flap repair. The patient commenced high-dose prednisolone and mycophenolate for FSGS. Surgery was delayed until renal function, albumin, and blood glucose levels were optimised. To address the anticipated challenges in healing, a laparoscopic loop colostomy was planned. 

**Figure 2:**
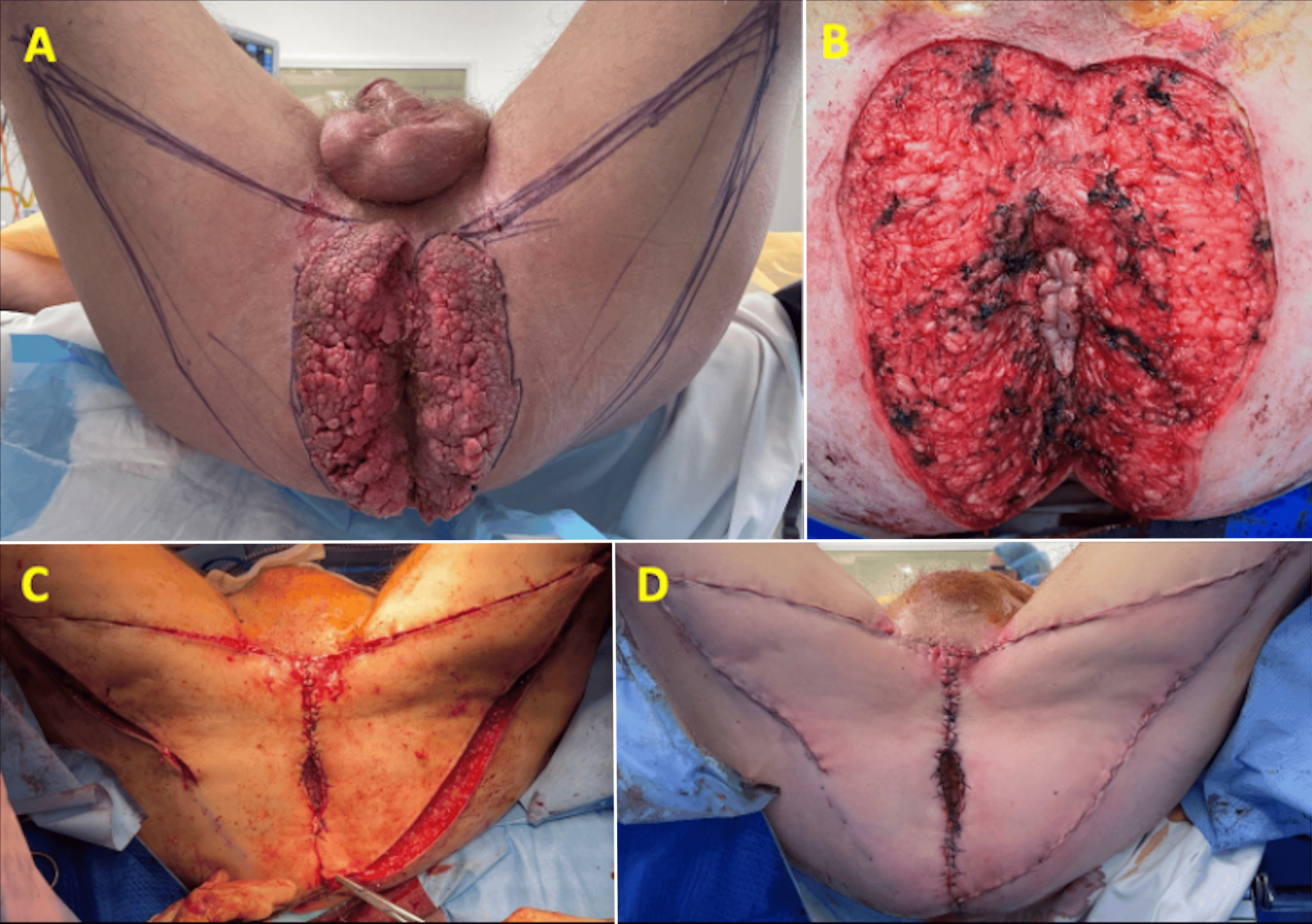
Intraoperative photographs demonstrating (A) the Buschke-Löwenstein tumour and marked advancement flaps with the patient in the modified Lloyd-Davies position, (B) the skin defect after the tumour was excised with preservation of the anoderm, (C) the right posterior thigh hatchet advancement flap and left V-Y advancement flap, and (D) the final appearance after the defect was reconstructed.

Under general anaesthesia, the patient was placed in the modified Lloyd-Davies position. The circumferential lesion extended 10 cm from the anal verge. Excision margins and advancement flaps were marked. The tumour was excised using monopolar and bipolar dissection, carefully preserving the anal sphincter complex. Bilateral posterior thigh fasciocutaneous advancement flaps were used for defect closure by the plastic surgery team (Figure 2). A loop sigmoid colostomy was created laparoscopically.

**Figure 3:**
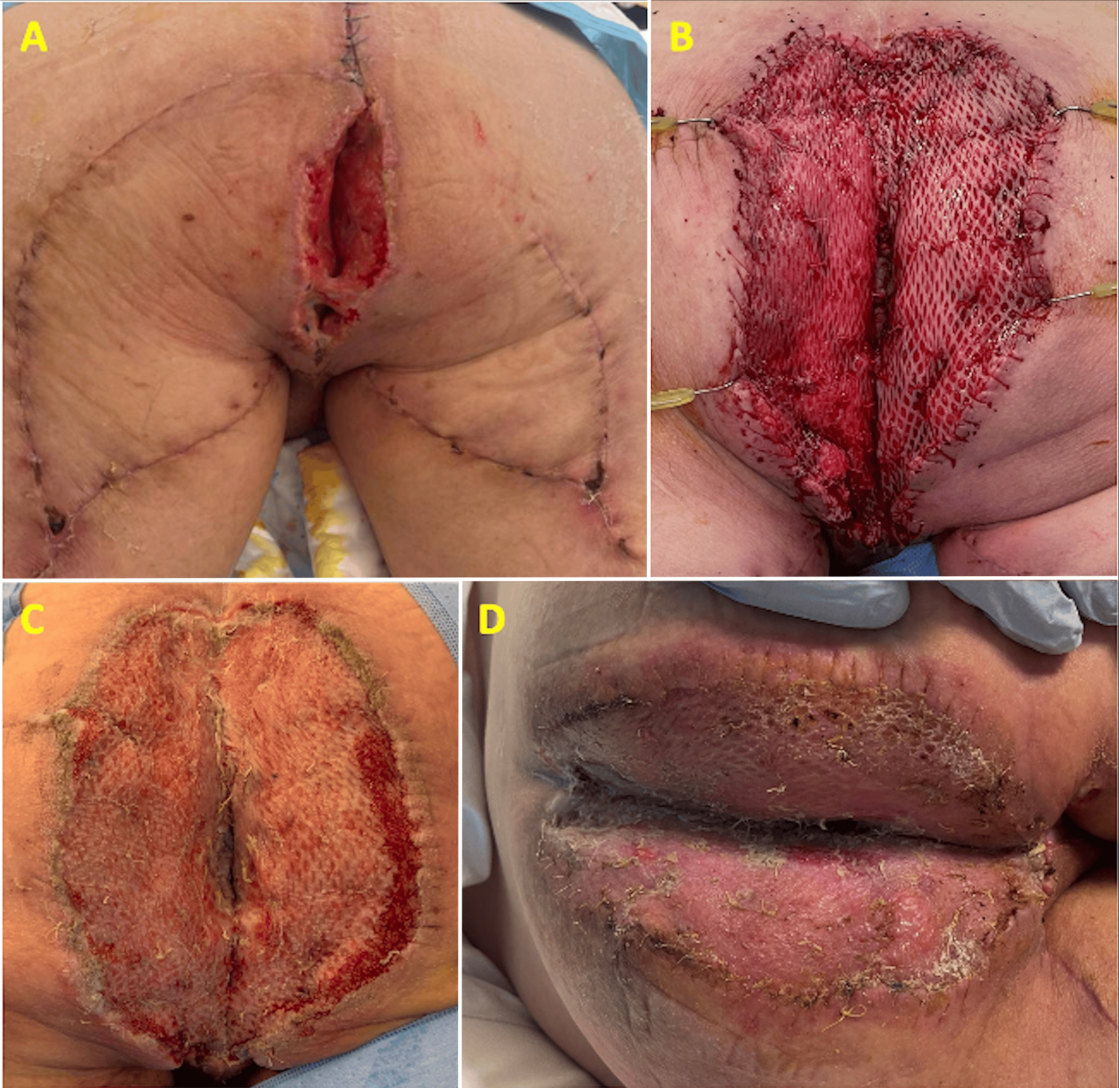
Progress photographs demonstrating (A) dehiscence of the flap around the perianal region with retraction of the anal canal, (B) inset of the split-thickness skin graft, and the appearance of the taken skin graft at (C) one week and (D) two weeks postoperative.

Postoperative care included daily dressing changes, stoma care, and bed rest with limited sitting to 30 degrees. Despite these measures, partial flap dehiscence and perianal retraction were noted two weeks postoperatively (Figure 3). The affected area was debrided and treated with a silver foam vacuum-assisted closure device. Following the formation of healthy granulation tissue, a split-thickness skin graft was applied, with most of the graft taken successfully.

Histopathology confirmed extensive condylomatous exophytic papillary growth consistent with a giant condyloma of Buschke-Löwenstein. A small 1.5 mm focus of early invasive squamous carcinoma was noted, with clear margins and no high-grade cytological atypia.

## Discussion

BLT is an uncommon clinical entity associated with HPV infection. Management is challenging due to disease progression, inconsistent treatment response, and high recurrence rates. Risk factors include immunodeficiency, diabetes, smoking, alcoholism, and multiple sexual partners [1,2]. In this case, the patient’s poorly controlled diabetes and history of lymphoma likely contributed to immunosuppression and BLT development.

Common symptoms include pain, bleeding, mass effect, purulent discharge, and dyschezia. BLTs carry a significant risk of malignant transformation, with reported rates of 50%-56% [1]. Transformation to squamous cell carcinoma (SCC), verrucous carcinoma, or basal cell carcinoma has been described [1]. While locally invasive, their metastatic potential remains limited, even with malignant transformation [2].

Surgical excision is the preferred first-line treatment, with evidence suggesting that clear surgical margins reduce recurrence risk [1,2,5]. Zhang et al. reported a recurrence rate of 23.7% amongst 38 patients during the median follow-up period of 23 months and 77% (7/9) of those with disease recurrence had positive resection margins [2]. On the other hand, clear surgical margins were achieved in 42% (16/38) of the cases, and only 12.5% (2/16) of those had disease recurrence [2]. Larger tumours (>20 cm) correlate with higher disease recurrence rates, as seen in reports by Kim et al. and Skowrońska-Piekarska et al. [6,7].

Surgical approaches should balance sphincter preservation, tissue reconstruction, and tumour eradication, with abdominoperineal resection (APR) reserved for extensive disease, malignant transformation or pelvic invasion [4,5]. Several case reports described surgical excision of BLT without skin reconstruction, out of which only one case noted the post-surgical complications of significant blood loss [8-13]. Multiple flaps have been described for reconstruction. These included fascio-cutaneous gluteal V-Y advancement flap, superior gluteal artery perforator (SGAP) flap, inferior gluteal artery perforator (IGAP) flap, autologous skin grafting from the thigh, and less commonly the vertical rectus abdominis myocutaneous (VRAM) flap reconstruction [5,7,14-18]. In this case, a bilateral posterior thigh fasciocutaneous advancement flap was used. 

Although not commonly described, colostomies have been performed in cases of extensive tumours or significant tissue invasion. Colostomies were performed in six case reports, either for those who underwent an APR [16,19], those with larger tumours (>20 cm) or extensive invasion involving the anus [6,12,17,20]. Notably, patients with colostomies were more likely to report postoperative complications of haemorrhagic shock, wound infection, abscesses, or the necrosis of tissue or graft. Other complications were observed in cases without colostomies, including wound dehiscence [18,21], abscess formation, and anal stenosis [7,14]. These were managed successfully by further surgical interventions such as wound debridement, anal dilatation, and sphincterotomy. The higher rates of significant complications associated with performing a colostomy in BLT resection suggest greater complexity and severity of disease as a poor prognostic factor. 

While diverting colostomy can aid wound healing and reduce contamination, it carries risks of additional complications, such as parastomal hernias and bowel obstructions [6,12,17,20]. A narrative review found that colostomy formation in Fournier’s gangrene could decrease the risks of further debridement, reduce the duration of wound healing, and facilitate the uptake of skin graft or flap [22]. Similarly, in patients with perineal pressure ulcers, those who underwent colostomy had lower ulcer recurrence rates and shorter healing times [23]. Patients with older age, malignancy or immunosuppression, were at higher risks of morbidity and mortality from stoma-associated complications. Judicious patient selection was recommended when deciding whether to use a colostomy [24]. Considering the age of our patient, and the extensive perineal wound (25 cm) at risk of faecal contamination, the benefits of a loop sigmoid colostomy in preventing faecal contamination and wound infection outweighed the risks. Despite the above attempt, the healing was complicated by the dehiscence of the flap, which required further debridement and reconstruction. This delay in wound healing was also evident in other cases [17,18,21].

The use of adjuvant therapies, such as radiation or chemotherapy with 5-fluorouracil (5-FU), mitomycin, or cisplatin, may be indicated in malignant transformation. Other minimally invasive treatments, such as intralesional 5-fluorouracil and cryotherapy, have shown limited success in smaller tumours (<10 cm) [1,4,6,25-27].

Given the lack of established guidelines in the treatment of BLT and indications for colostomy, our case highlights the challenges in managing BLT and its postoperative complications, especially in a patient with complex medical comorbidities in a rural setting. Limited access to specialised surgical and multidisciplinary care may further delay diagnosis and treatment of rare tumours like BLT, underscoring the systemic challenges in care coordination and reconstructive planning for complex cases [28].

## Conclusions

Buschke-Löwenstein tumours are rare and challenging to manage due to high recurrence rates and complex wound care needs. Radical excision with clear margins remains the cornerstone of treatment. Our experience underscores the importance of a multidisciplinary approach in managing this rare condition, with such collaborative efforts between the renal and endocrine specialists, colorectal and plastic surgical teams, wound and stoma care, and general practitioners for follow-up in the community. However, optimal management requires individualisation based on lesion characteristics, patient factors and available resources. This case illustrates the role of pre-operative medical optimisation and various surgical techniques in achieving the satisfactory oncological, functional and cosmetic outcomes.
